# Characterising demographics, knowledge, practices and clinical care among patients attending sickle cell disease clinics in Eastern Uganda

**DOI:** 10.12688/wellcomeopenres.15847.2

**Published:** 2020-07-07

**Authors:** Peter Olupot-Olupot, Ham Wabwire, Carolyne Ndila, Ruth Adong, Linus Ochen, Denis Amorut, Grace Abongo, Charles B. Okalebo, Sarah Rachael Akello, Joy B. Oketcho, William Okiror, Sarah Asio, Amos Odiit, Florence Alaroker, Gideon Nyutu, Kathryn Maitland, Thomas N. Williams

**Affiliations:** 1 Mbale Clinical Research Institute, Mbale, Uganda; 2 Faculty of Health Sciences, Busitema University, Mbale, Uganda; 3 Soroti Regional Referral Hospital, Soroti, Uganda; 4 Atutur District Hospital, Atutur, Uganda; 5 Ngora Freda Carr Hospital, Ngora, Uganda; 6 KEMRI-Wellcome Trust Research Programme, Kilifi, Kenya; 7 Faculty of Medicine, Imperial College London, London, UK

**Keywords:** Sickle Cell Disease, knowledge, care, wellbeing, Eastern Uganda

## Abstract

**Background**: In Uganda to date, there are neither established registries nor descriptions of facility-based sickle cell disease (SCD) patient characteristics beyond the central region. Here, we summarize data on the baseline clinical characteristics and routine care available to patients at four clinics in Eastern Uganda as a prelude to a clinical trial.

**Methods**: Between February and August 2018, we conducted a cross-sectional survey of patients attending four SCD clinics in Mbale, Soroti, Atutur and Ngora, all in Eastern Uganda, the planned sites for an upcoming clinical trial (H-PRIME: ISRCTN15724013). Data on socio-demographic characteristics, diagnostic methods, clinic schedules, the use of prophylactic and therapeutic drugs, clinical complications and patient understanding of SCD were collected using a structured questionnaire.

**Results**: Data were collected on 1829 patients. Their ages ranged from 0 to 64 years with a median (IQR) of 6 (3-11) years. 49.1% of participants were male. The majority (1151; 62.9%) reported a positive family history for SCD. Approximately half knew that SCD is inherited from both parents but a substantial proportion did not know how SCD is transmitted and small numbers believed that it is acquired by either transfusion or from other people. Only 118/1819 (6.5%) participants had heard about or were using hydroxyurea while 356/1794 (19.8%) reported stigmatization. Participants reported a median of three (IQR 1-4) hospital admissions during the preceding 12 months; 80.8% had been admitted at least once, while 14.2% had been admitted more than five times. Pain was the most common symptom, while 83.9% of those admitted had received at least one blood transfusion.

**Conclusion:** The majority of patients attending SCD clinics in Eastern Uganda are children and few are currently being treated with hydroxyurea. The data collected through this facility-based survey will provide background data that will be useful in planning for the H-PRIME trial.

## Introduction

Following years of neglect in many parts of sub-Saharan Africa (SSA), sickle cell disease (SCD) is increasingly being recognized as a disease of major public health significance ^1^. Nevertheless, the lack of universal newborn screening, SCD registries and SCD surveillance programmes ^2^ mean that accurate data on the total numbers living with the disease and its clinical consequences remain rather limited. Globally, approximately 330,000 children are born with the most common form of SCD (HbSS) every year ^3^, the majority (>80%) in SSA where carrier frequencies of 10–20% are common, and up to 2% of all births are affected ^3^. Typically, SCD causes chronic ill health that is interposed by acute complications, which result in cumulative damage to multiple end-organs ^4^. It has been estimated that in many parts of SSA between 5 and 16% of all under-five mortality is attributable to SCD ^5, 6^.

Uganda is ranked as the country with the fifth highest birth rate of children with SCD globally and the highest in Eastern Africa. Throughout much of the country, the carrier frequency is between 9 and 21% ^7^ and an estimated 10–15,000 babies are born with SCD every year ^3, 7^. Nevertheless, few facility-based studies have been published that describe the characteristics of cases, their clinical care or patterns of disease awareness in Uganda. In the current study, we present the results of a descriptive study that documents these parameters from SCD clinics within the Eastern Region of the country in preparation for an upcoming clinical trial ^8^ that will investigate potential options for the pragmatic treatment of SCD in low resource settings.

## Methods

### Ethical statement

Ethical approval for the study was obtained from the Mbale Regional Referral Hospital Research & Ethics Committee (MRRH-REC; approval number MRRH-REC IN-COM 007/2018) while written permission to conduct the survey was also obtained from each of the health facilities individually. Written informed consent to administer the questionnaire was obtained from all participants or their parents who were approached directly and individually in the study clinics. Paticipants were also informed about the plans for data sharing and publication. The Mbale Clinical Research Institute, a research entity affiliated to the Uganda National Health Research Organization (UNHRO), permits the publication of this manuscript.

### Study design and site

Our study was conducted in the Elgon and Teso sub-regions of Eastern Uganda where the prevalence rates of SCD is high ^7^. There are 24 district level health facilities within these regions that offer general care for all patients, including subjects with SCD. However, only five of these facilities have dedicated SCD clinics. In the current study, we collected data from four of these clinics, one in Elgon and three in the Teso sub regions, selected on the basis that all will contribute participants for the upcoming H-PRIME trial. Between February and August 2018, we conducted a cross-sectional survey at our four target clinics at (1) Mbale Regional Referral Hospital; (2) Atutur District Hospital; (3) Ngora Health Centre IV; and (4) Soroti Regional Referral Hospital. Details of these government-aided not-for-profit, charge-free facilities are summarized in the Supplementary Table (see *Extended data*) ^9^. Following a process of informed consent, all patients attending the SCD clinics held at these hospitals on survey days were invited to participate in the survey. Each was approached individually by a member of the study team who administered the consenting materials and sought individual informed consent. The only inclusion criteria were attendance at one of the study clinics on one of the surveillance days plus consent to participate while any patient declining consent was excluded. Before finalizing, the questionnaire was first piloted in a small group of SCD patients with their families who were attending the SCD clinic within the four health facilities. The pilot involved testing the wording, the order of questions, the range of possible answers and the clarity of the instructions for both the whole questionnaire and the individual questions.

### Data collection

A questionnaire was designed to address a range of issues including the socio-demographic characteristics of patients, the method of diagnosis, the frequency of clinic attendance, knowledge and understanding of SCD, the use of therapeutic and prophylactic drugs and the frequency of SCD-specific crises, inpatient admissions and blood transfusions. In addition, data were collected on disease complications, and awareness of hydroxyurea (see *Extended data*) ^9^. The questions on the study questionnaire were read to the study participants by study personnel in their preferred language, who then completed the questionnaire on behalf of study participants. Responses were deferred to parents or carers of those <18 years and to the participant themselves if ≥18 years. The whole process took an average of 20–30 minutes / participant interviewed.

### Statistical analysis

The sampling approach was pragmatic, and no sample-size calculation was considered. Data were analyzed using the R statistical package V3.6.0 ^10^. Categorical variables were analyzed as frequencies, while continuous variables were computed as medians with interquartile ranges (IQR). Participants were stratified into the age groups ≤5 years and >5 years. Between-group differences were assessed using the χ ^2^ test. P-values of <0.05 were considered significant.

## Results

While clinic records were not computerised and were somewhat incomplete, the total number of patients who were registered at these 4 SCD clinics was 2257. When conducting the survey, we noticed that some of the clinics had registered fewer patients than they were actually handling. All 1829 patients who were in attendence during the study period and who were invited to participate gave their consent and were included in the study, so at minimum, 428 (19%) of the registered participants did not attend their clinics during the course of the survey.

### Characteristics of respondents

The social and demographic characteristics of the 1829 study participants are summarized in Table 1. Their age range was 0 to 64 years with a median (IQR) of 6 (3–11) years, while 858/1748 (49.1%) were females ^9^. More than half (1006/1820; 55.3%) were >5 years, only 371 (20.3%) being children <2 years and 92 (5.1%) being adults >18 years. Three of the four study sites were located in the Teso sub-region of Eastern Uganda and as a result, the majority of participants (1319/1826; 72.2%) were Iteso, followed by 204 (11.2%) from the Bagisu ethnic group, the balance being Kumam, BagwereBagwere and others. The majority of subjects (1588/1782; 89.1%) were Christian, while 155 (8.7%) and 39 (2.2%) were either Muslim or from other religions, respectively. A majority (1151; 62.9%) had another family member with SCD, of whom 298 (16.3%) had died. Of these deaths, 69.9% were among children <5 years old. Around half (920/176; 52.2%) of participants reported visiting the SCD clinic at least once every month and 629/1761 (35.7%) at least once every 3 months. A total of 143/1761 (8.1%) reported that they had never previously attended the SCD clinic for their treatment or care.

**Table 1.** Socio-demographic characteristics of respondents.

Characteristic	N (%)
**Gender**	
Female	858 (49.1)
**Age**	
≤5 years	814 (44.7)
>5 years	1006 (55.3)
**Tribe**	
Iteso	1319 (72.2)
Gishu	204 (11.2)
Kumam	114 (6.2)
Bagwere	78 (4.3)
Others	111 (6.1)
**Religion**	
Christians	1588/1782 (89.1)
Muslims	155/1782 (8.7)
Others	39/1782 (2.2)
**Family history**	
Relative with SCD	1151/1829 (62.9)
Siblings died of SCD	298/1829 (16.2)
**Self-reported clinic visits**	
Never	143/1761 (8.1)
Bi-weekly	21/1761 (1.2)
Monthly	920/1761 (52.2)
Bi-monthly	39/1761 (2.2)
Every 3 months	629/1761 (35.7)
Every 6 months	1/1761 (0.1)
Once a year	8/1761 (0.5)
**Self-reported** **hospitalization in the** **past 12 months**	
0	327/1707 (19.2)
1	367/1707 (21.5)
2	290/1707 (17.0)
3	252/1707 (14.8)
4	131/1707 (7.7)
5	97/1707 (5.7)
>5	243/1707 (14.2)

SCD, sickle cell disease.

### Hospitalizations

The median number of hospitalizations within the preceding twelve-month period was three (IQR 1-4). Of 1707 participants with data on hospitalization, 1380 (80.8%) had been admitted at least once, while 243 (14.2%) had been admitted more than five times ( Table 1). The most common symptoms at admission are summarized in Table 2. Overall, pain was present in 1261 of 1342 (91.8%) admissions, while other common syndromes included severe anaemia, hand-foot syndrome and malaria. In general, patterns of clinical illness were similar across age-groups, although hand-foot syndrome and pneumonia were significantly more common ( *P*<0.0001), while pain was significantly less common (p=0.0002) among children <5 years old. Of the participants who had been admitted to hospital during the preceding year, 951/1133 (83.9%) had received at least one blood transfusion, while 151 (13.3%) had received five or more ( Table 3).

**Table 2.** Features present during the most recent hospitalization among respondents who were admitted within the preceding 12 months.

Clinical feature *	Overall, N (%)	≤5 years (%)	>5 year (%)	P-value
Totals	1374	656	718	n/a
Painful crisis	1261 (91.8)	583 (88.8)	678 (94.4)	0.0002
Fever	949 (69.1)	463 (70.5)	486 (67.6)	0.24
Anaemia	852 (62.0)	409 (62.3)	443 (61.6)	0.80
Hand foot syndrome	697 (50.7)	391 (59.6)	306 (42.6)	<0.0001
Malaria	622 (45.3)	288 (43.9)	334 (46.5)	0.33
Convulsions	20 (1.5)	11 (1.6)	9 (1.2)	0.42
Very large spleen	83 (6.0)	39 (5.9)	44 (6.1)	0.88
Bone infection	22 (1.6)	11 (1.7)	11 (1.5)	0.83
Pneumonia	109 (7.9)	73 (11.1)	36 (5.0)	<0.0001
Stroke	25 (1.8)	9 (1.4)	16 (2.2)	0.23

* Clinical features are self-reported rather than being based on hospital records. Most participants reported more than one clinical syndrome.

**Table 3.** The number of blood transfusions received by study participants during the preceding 12 months.

Number of blood transfusions (self reported)	Totals	Age in years		P-value
		≤5	>5	
	N (%)	n (%)	n (%)	
0	182 (16.1)	86 (16.1)	96 (16.0)	0.45
1	352 (31.0%)	194 (36.3)	158 (26.4)	0.05
2	235 (20.7%)	117 (21.9)	118 (19.7)	0.94
3	137 (12.1%)	59 (11.0)	78 (13.0)	0.10
4	76 (6.7%)	34 (6.4)	42 (7.0)	0.35
5	38 (3.5%)	11 (2.1)	27 (4.5)	0.009
>5	113 (9.8%)	33 (6.2)	80 (13.4)	0.0004

### Knowledge of SCD

The perceptions of participants with regard to SCD are summarized in Table 4. More than half (934/1729; 54.0%) understood that SCD is inherited from both parents although small proportions believed that it was acquired through either a blood transfusion (17; 1%) or through personal contact (3; 0.2%). Almost half of all participants (775; 45%) had no understanding of how the disease is acquired. The metabisulphite sickling test and haemoglobin electrophoresis were reported as the two most common methods of diagnosis. Feelings of stigmatization were reported by 356/1794 participants (19.8%). ****

**Table 4.** Participant knowledge of sickle disease and awareness of hydroxyurea.

Question	N (%)
**What is the cause of SCD?**	
Inherited	934/1729 (54.0)
Acquired from blood transfusion	17/1729 (1.0)
Acquired from body contact	3/1729 (0.2)
Don’t know	775/1729 (44.8)
**Have you heard about hydroxyurea?**	
No	1711/1829 (93.5)
Yes	118/1829 (6.5)
Patient or a family member are using hydroxyurea	41/1829 (2.2)
**If using hydroxyurea, where do you get the drug?**	
Private pharmacy	15/41(36.5)
Private clinic/hospital	13/41(31.7)
Projects	4/41 (9.7)
Government hospital	0/41 (0)

SCD, sickle cell disease.

### Awareness and use of hydroxyurea

In total, 118/1829 (6.5%) of study participants had heard about hydroxyurea. None were taking it personally but 41 (2.2%) reported that a relative was using it ( Table 4). The main sources of hydroxyurea for relatives using the drug were either private clinics or community pharmacies ( Table 4).

### Antibiotics and immunization

The routine treatments received by participants are summarised in Table 5. It was found that 777/1820 participants (42%) were receiving antimalarial prophylaxis with sulphadoxine/pyrimethamine, the current standard of care for children with SCD in Uganda ^11^, while 940 (51.6%) were taking chloroquine. Overall rates of immunization with BCG, measles, polio and DPT vaccines were >90%, while that for the PCV10 pneumococcal vaccine was 626/1820 (34.4%). A significant proportion of those >5 years, in whom penicillin prophylaxis is not part of the Ugandan standard of care ^11^, were receiving either oral penicillin-V (105/1006; 10.4%) or intramuscular benzathine penicillin (49/1006; 4.9%).

**Table 5.** Treatment and immunization by age group.

Treatment	Overall, N (%)	Age group	
		≤5 years (%)	>5 year (%)
Total	1820 (100)	814 (100)	1006 (100)
**Antimalarial prophylaxis**			
Sulphadoxine/pyrimethamine	777 (42.7)	340 (41.8)	437 (43.4)
Chloroquine	940 (51.6)	399 (49.0)	541 (53.8)
Proguanil	2 (0.1)	0 (0.0)	2 (0.2)
**Use of antibiotics**			
Penicillin V	211 (11.6)	106 (13.0)	105 (10.4)
Benzathine Penicillin	155 (8.5)	106 (13.0)	49 (4.9)
**Immunizations**			
BCG	1796 (98.7)	805 (98.9)	991 (98.5)
Pneumococcal vaccine	626 (34.4)	422 (51.8)	204 (20.3)
Measles	1742 (95.7)	765 (94.0)	977 (97.1)
Polio	1568 (86.2)	700 (86.0)	868 (86.3)
DPT	1770 (97.3)	789 (96.9)	981 (97.5)

### Laboratory capacity

Tests that were commonly conducted on SCD patients at the study facilities are summarised in Table 6. Varying levels of laboratory capacity were available at these health facilities for investigations related to the management of SCD. For example, although all had the capacity to perform complete blood counts and tests for malaria, only Mbale and Soroti Regional Referral Hospitals were able to conduct clinical chemistry assays such as renal and liver function tests. Neither Mbale nor Soroti Regional Referral Hospitals had facilities for measuring levels of haemoglobin F (HbF), but the other sites had access to HbF measurements through referral to the Central Public Health Laboratory in Kampala. None of the clinics had direct access to haemoglobin electrophoresis. Whenever laboratory services were not available, patients at all four clinics were sent to private laboratories.

**Table 6.** Laboratory tests available by facility.

Laboratory tests commonly performed	Facility			
	Mbale	Soroti	Atutur	Ngora
**Haematology**				
Complete blood count	No	No	Yes	Yes
Haemoglobin	No	No	Yes	Yes
Red cell indices	No	No	Yes	No
Haemoglobin F concentration	No	No	Yes	Yes
Reticulocyte count	No	No	Yes	No
**Sickle diagnostics**				
Hb electrophoresis/analysis	Yes	No	Yes	No
Sickling test	Yes	No	No	Yes
**Malaria diagnosis**				
Peripheral blood film	Yes	Yes	Yes	Yes
Rapid diagnostic tests	Yes	Yes	Yes	Yes
Serum biochemistry tests	Yes	Yes	Yes	Yes
**Microbiology**				
Blood culture	Yes	Yes	No	No
Culture and sensitivity	Yes	Yes	No	No

## Discussion

Through this survey we have investigated the demographics, knowledge, practices and a range of factors relating to clinical care of patients attending four SCD clinics in Eastern Uganda as a prelude to the upcoming H-PRIME clinical trial ^8^. All four clinics are in an area with a high prevalence of SCD ^7^ and have treated patients with SCD for many years.

In contrast to clinics in Europe and North America, where the frequency of attendance at SCD clinics is typically tailored to patient needs ^12^, more than half of respondents were attending clinic on a monthly basis. To the best of our knowledge, there have been no evaluations of the relative effectiveness of different clinic schedules. Although transport costs have been cited as a potential barrier to adherence ^13^, more than 90% of patients at the Mbale clinic comply with their monthly visits (PO-O, unpublished report). Nevertheless, it seems likely that such regular attendance must represent a significant challenge for patients, their families and the medical staff involved and that if safe, a less frequent schedule would be of benefit to all.

The poor understanding by participants of even some of the most basic facts about SCD, including its mode of acquisition, was striking. Almost half of respondents did not know how the disease is acquired, and small proportions believed that it could be caught through either blood transfusion or personal contact. Similarly, many participants reported that they felt stigmatized, a finding that accords with observations from other studies ^14– 16^. The need for education about SCD, both in these clinics and within the wider society, are clear priorities for these clinics going forwards ^17^.

Previous studies have highlighted the high rate of mortality within Africa among children with undiagnosed SCD, with one suggesting that historically this may have been as high as 90% in those <5 years ^18^. While a large proportion of the patients attending these SCD clinics were <5 years old, the age range of respondents was wide (0 to 64 years), and almost half were >5 years old. This observation supports one recent study, conducted in Kenya ^19^, which concluded that mortality can be substantially improved by the introduction of just a handful of simple interventions that include education and measures to prevent both malaria and bacterial infections. Nevertheless, it is also clear from our survey that irrespective of improved survival, many respondents were facing significant health challenges, exemplified by a high rate of admission to hospital. More than 80% had been admitted to hospital in the preceding year and almost one fifth had been admitted five times or more. Pain was a common feature during those admissions, occurring at a substantially higher prevalence than that reported in several recent studies ^20, 21^. While the reason for this difference is unknown, we hypothesize that it might reflect socio-economic issues and the local epidemiology of conditions such as bacterial diseases or malaria. A further observation was the high rate of transfusion among study participants. More than 80% of those who had been admitted to hospital during the preceding year had received at least one blood transfusion, and 13.3% had received five transfusions or more. This reinforces the message from a number of recent studies that have reported the heavy demand that patients with SCD place on blood transfusion services in a number of African settings ^19, 22– 24^. For example, in the recently reported multi-centre Transfusion and Treatment of Severe Anaemia in African Children Trial (TRACT), 33% of those recruited with severe and complicated anaemia had SCD, of whom almost half had not been previously diagnosed ^23, 24^.

Our study had a number of limitations, not least the fact that our questionnaire was administered over a limited period of time, meaning that it was not possible for us to capture questions such as the seasonal patterns of specific complications. Nevertheless, taken together, the results of our survey suggest that many of the participants could potentially stand to benefit from access to disease-modifying therapies. Of those that are commonly available, all are either too complex to administer or too expensive to be realistic options, with one exception - hydroxyurea. Hydroxyurea is an orally-administered drug that is included for the treatment of SCD in the WHO list of essential medicines for children ^25^. Where it has been used in resource-rich countries with regular monitoring, the safety record of hydroxyurea has been excellent ^26^. Nevertheless, experience in the use of hydroxyurea in Africa is limited, where a number of potential safety concerns have been raised ^27^. Perhaps most importantly, one of the mechanisms of action is through the induction of some degree of immunosuppression. That necessitates the careful supervision of treatment and regular laboratory monitoring when hydroxyurea is used. In two recent studies it has been shown that hydroxyurea can be safely used in Africa when accompanied by regular laboratory monitoring ^28, 29^. However, our current survey shows that the laboratory services that are currently available at our study sites would not be adequate to support the use of hydroxyurea in a similar way. Indeed, not all clinicians even have access to reliable diagnostic tests and as a result, we cannot even be sure that all those attending these clinics definitely have SCD. In Uganda, most patients with SCD live in rural settings where both laboratory capacity and specialist support are limited. In our survey, none of the patients were receiving hydroxyurea, although a small proportion had a relative who was using it. Further studies that evaluate the use of hydroxyurea in resource-poor settings such as these - a major aim of the H-PRIME trial ^8^ - are urgently needed.

Finally, our study shows that the standards of care, as laid out in The Uganda Clinical Guidelines 2016 ^11^, with regard to the prevention of infections in children with SCD are not being followed systematically. While these guidelines recommend the use of daily penicillin V for bacterial prophylaxis in children <5 years and monthly sulphadoxine/pyrimethamine for the prevention of malaria ^11^, we found that a high proportion of older participants were still taking penicillin and that more than half were using chloroquine instead of sulphadoxine/pyrimethamine. Further studies regarding the optimal treatments for both are also needed.

## Conclusions

Our survey illustrates some of the problems faced by those affected by SCD, and by their health-care providers. Although the results of our study should be interpreted with caution, given that many of our observations relied on patient recall rather than observed events, they do allow us to make some important deductions. First, patient and caregiver knowledge of SCD within this cohort was very low. Patient and caregiver knowledge of SCD is low. There is high morbidity in SCD patients represented by frequent clinic visits, frequent admissions, and frequent blood transfusions. Hydroxyurea is not in use in the setting of this study, yet it has been found to reduce morbidities in SCD. There remains much to be done to improve the lives of people living with SCD in low income countries. Locally appropriate interventions need to be based on research conducted locally, the central aim of the H-PRIME trial ^8^.

## Data availability

### Underlying data

Harvard Dataverse: Data for: Characterising demographics, knowledge, practices and clinical care among patients attending sickle cell disease clinics in Eastern Uganda. https://doi.org/10.7910/DVN/AIDN0V ^9^.

This project contains the following underlying data:

- SCD_Manuscript_data26042020.xlsx (questionnaire answers and sociodemographic characteristics for all participants)
- SCD_Manuscript_data dictionary.tab (data dictionary)

### Extended data

Harvard Dataverse: Data for: Characterising demographics, knowledge, practices and clinical care among patients attending sickle cell disease clinics in Eastern Uganda. https://doi.org/10.7910/DVN/AIDN0V ^9^.

- Sickle cell questionnaire.pdf
- SCD Informed Consent Form.df
- Extended data.docx (Supplementary Table)

Data are available under the terms of the Creative Commons Zero "No rights reserved" data waiver (CC0 1.0 Public domain dedication).
