# Supplementary material for: Characterising demographics, knowledge, practices and clinical care among patients attending sickle cell disease clinics in Eastern Uganda
Source: Wellcome Open Res. 2020 Jul 7;5:87. Originally published 2020 May 4. [Version 2] doi: 10.12688/wellcomeopenres.15847.2 (PMC7406951; doi:10.12688/wellcomeopenres.15847.2)
Supplement: Peer review history

# Peer review: Reviewer response for version 2

**DOI:** 10.21956/wellcomeopenres.17639.r39440

**Published:** 2020-08-05

**Authors:** Baba Inusa

**Affiliations:** 1 Department of Paediatric Haematology, Evelina London Children's Hospital, Guy's and St Thomas NHS Foundation Trust, London, UK

I am happy that this paper reads a lot better and it highlights the real life scenario looking at a less urban setting where the majority of people with sickle cell may seek medical attention. It is also the case that patients in these settings may also attend the health facility because of a medical emergency rather than just a routine clinic visit follow up. It is not surprising that pain is such a prevalent feature.

 It is worth noting the high proportion who indicated they do not know the cause of sickle cell disease. This is a short survey and therefore did not have the capacity to explore further. It would be interesting if the respondents were able to indicate their own hypotheses about possible causes. This would be an important entry point for education and awareness. SCD in Africa calls for community based programmes to address the not only the diagnosis but address myths as much as the introduction of penicillin V and hydroxyurea therapy

 It is not possible to address the issue of survival from this survey, I do not think the study design and implementation supports that in any way. It is, however, interesting that a high proportion of patients above age 5 were surveyed. 

 it is also difficult to be certain about the diagnosis based on solubility test alone and probably based on clinical features alone. While this may be correct in the majority of cases the risk is that some individuals with HbAS may pass as SCD and may in fact be living with a presumed diagnosis while some with SCD may also be thought to be sickle cell trait (HBAS). This calls for urgency improvement in diagnostic capacity of a community hospital or at least a twinning with the specialist centers. 

 The authors may wish to say something about the verification of the diagnosis, whether all these cases were actually SCD.

Is the work clearly and accurately presented and does it cite the current literature?

Yes

If applicable, is the statistical analysis and its interpretation appropriate?

Partly

Are all the source data underlying the results available to ensure full reproducibility?

Yes

Is the study design appropriate and is the work technically sound?

Partly

Are the conclusions drawn adequately supported by the results?

Partly

Are sufficient details of methods and analysis provided to allow replication by others?

Partly

Reviewer Expertise:

Sickle cell disease including global haematology work I am particulary intereted in Africa and currently lead projects in Africa.

I confirm that I have read this submission and believe that I have an appropriate level of expertise to confirm that it is of an acceptable scientific standard.

# Peer review: Reviewer response for version 2

**DOI:** 10.21956/wellcomeopenres.17639.r39439

**Published:** 2020-07-07

**Authors:** Simon Dyson

**Affiliations:** 1 Unit for the Social Study of Thalassaemia and Sickle Cell, School of Allied Health Sciences, De Montfort University, Leicester, UK

No further comments

Is the work clearly and accurately presented and does it cite the current literature?

Yes

If applicable, is the statistical analysis and its interpretation appropriate?

I cannot comment. A qualified statistician is required.

Are all the source data underlying the results available to ensure full reproducibility?

Yes

Is the study design appropriate and is the work technically sound?

Yes

Are the conclusions drawn adequately supported by the results?

Yes

Are sufficient details of methods and analysis provided to allow replication by others?

Yes

Reviewer Expertise:

Sociologist

I confirm that I have read this submission and believe that I have an appropriate level of expertise to confirm that it is of an acceptable scientific standard.

# Peer review: Reviewer response for version 1

**DOI:** 10.21956/wellcomeopenres.17383.r38631

**Published:** 2020-05-11

**Authors:** Baba Inusa

**Affiliations:** 1 Department of Paediatric Haematology, Evelina London Children's Hospital, Guy's and St Thomas NHS Foundation Trust, London, UK

This is an interesting paper. It is very relevant to describe the context of the proposed H-PRIME study. It is also important to describe the current situation in the areas where sickle cell services are lacking. It seems to be ambitious to administer such wide-ranging questions to the respondents within 20-30 minutes. More time would be needed if further work is required in order to introduce education sessions for the clients. It is not clear how the information about diagnostic methods was collected. Was the sodium metabisulphite test used as diagnostic or screening or confirmatory following initial testing, in which case it could be used to confirm the variant haemoglobin as HbS? It is questionable to rely on the solubility test for diagnosis. How do you know that these are all sickle cell disease patients especially for those in Soroti locations?

 The limitation of research based at a health facility was mentioned in the introduction, yet this work seems to have done exactly that. The fact that the sample was not done all year round may have missed important patter of clinic attendance.

 Some of the key messages were not supported by references - frequency of clinics in the developed countries as stated between 3-6 monthly. The clinic frequencies are usually related to the therapies that patients are receiving e.g.Hydroxyurea which requires regular follow up and both UK and NIH management do have management guidelines with varying degrees of supportive evidence.

 I can not see the socioeconomic information, if any was collected.

 It is desirable to provide more information on the reason for high rate of blood transfusion in his patient group

 The language generally could be improved.

Is the work clearly and accurately presented and does it cite the current literature?

Yes

If applicable, is the statistical analysis and its interpretation appropriate?

Partly

Are all the source data underlying the results available to ensure full reproducibility?

Yes

Is the study design appropriate and is the work technically sound?

Partly

Are the conclusions drawn adequately supported by the results?

Partly

Are sufficient details of methods and analysis provided to allow replication by others?

Partly

Reviewer Expertise:

Sickle cell disease including global haematology work I am particulary intereted in Africa and currently lead projects in Africa.

I confirm that I have read this submission and believe that I have an appropriate level of expertise to confirm that it is of an acceptable scientific standard, however I have significant reservations, as outlined above.

# Peer review: Reviewer response for version 1

**DOI:** 10.21956/wellcomeopenres.17383.r38633

**Published:** 2020-05-05

**Authors:** Simon Dyson

**Affiliations:** 1 Unit for the Social Study of Thalassaemia and Sickle Cell, School of Allied Health Sciences, De Montfort University, Leicester, UK

The study is a cross-sectional (2018) descriptive survey of sickle cell disease (SCD) patients, principally children across four clinics in Eastern Uganda, encompassing description of reported symptoms, hospitalizations, patient knowledge of SCD and laboratory capabilities of the four SCD clinics. It describes the reported use of and awareness of the drug hydroxyurea (hydroxycarbamide) prior to implementation of a clinical trial of hydroxyurea in that setting.

 Overall, the study is soundly conducted and meticulously reported and with a few minor amendments is ready to be published in my view. It could very readily be published “as-is” so my notes are suggestions rather than requirements.

 My comments are as a sociologist and so may, therefore, differ in content and focus from those of medical reviewers.

  

1. A major tension between disciplines is the widespread medical/public health/WHO use of the term “burden” (line 6 and passim). To a sociologist, this reads as disabling (discrimination in terms of disability rights). It is arguably a short step from recognizing the challenge of SCD to the person living with SCD, and/or the challenge to the health services of a country, to say that the person’s disease is a burden to them and/or that they are a burden on the health services of their country. The authors later note the stigmatization that can affect people living with SCD and, to a sociologist, the use of burden in the medical lexicon is part of that very stigmatization. Is there not a less pejorative way in which the ideas might be expressed? For example, the person living with SCD might be said to “face challenges of living with SCD” rather than have the burden of SCD. SCD may create pressure for health services or demand for health services rather than be a burden on health services.
2. In paragraph 2, Introduction would it be an idea to tell the reader if the four SCD clinics represent ALL the clinics in eastern Uganda or are there others not included (or are there other hospitals in the region without an SCD clinic?) 
3. Given that the participants were mainly children, the methods should ideally report that the questionnaire was read to a parent/carer/person accompanying the young person. This then raises the question of at what age did the researchers defer to the answer of the person with SCD themselves rather than to an accompanying person?  
4. In paragraph 1 of Results, might it be possible to know what per cent of all clinic patients with SCD registered at the respective clinics are represented by the pragmatic sample recruited?
5. In terms of economy of presentation the authors present Male per cent. Again just to note that in terms of challenging sexism in official statistics (Ann Oakley, circa 1979) sociologists have encouraged reporting of female statistics before male statistics. It would be possible here to report female per cent and leave male per cent implied rather than the other way around?  
6. For most of the data, it is not a leap of faith to record reported answers (tribe, religion) as representing the actuality. However, where other potential evidence exists (in clinic or hospital records) I think it would be wise to emphasise that some information is *reported* information rather than hospital records? This would apply to the number of hospitalizations, the number of blood transfusions, use of penicillin/malaria prophylaxis etc, and even to the regularity of clinic visits. Presumably, there might be some discrepancies if answers provided to a structured interview were compared with hospital records?  
7. There may not be space to open this issue up but presumably the skewing of the clinic attendees to young children tells us something about the age structure of the SCD population in rural Uganda (always presuming numerous older SCD people are not living without any contact with health services, which seems highly unlikely). It may have to wait for another paper but presumably, a comparison could be made between the age structure of SCD clinic attendees and all hospital attendees? In the absence of newborn SCD screening this could tell us something about under-fives SCD mortality in Uganda before implementation of treatment with hydroxyurea?
8. The fact that symptoms appear to be reported symptoms rather than medically documented symptoms may be the reason the authors do not comment more on Table 2. Two classic child-focussed symptoms of SCD I am aware of are splenic sequestration and hand-foot syndrome. Given high rates of the latter and low level of reported enlarged spleens, I wonder if this is indicative of high mortality from splenic sequestration in the under-fives? The reported levels of stroke are very low but I don’t know if blood transfusions are being used to manage this?  
9. In Table 6, it is noted that only two of the four centres have the capability to undertake haemoglobin electrophoresis. This may be my lack of knowledge but other than the sickle cell solubility test (which does not distinguish HbSS, HbAS and compound heterozygous forms of SCD?) how do we know the people in the study definitively had SCD? Perhaps the diagnosis is based on sickle cell solubility test plus characteristic SCD symptoms? Or is the range of tests available at any one centre sufficient to be confident in an SCD diagnosis?
10. With regard to stigma, the authors may care to look at https://doi.org/10.1016/j.socscimed.2016.05.029 for two reasons. (1) To understand what stigma is and is not and (2) To see ways in which stigma can be challenged through research as well as described in research.
11. Page 7, fourth paragraph: higher prevalence of pain is noted compared to studies in Saudi Arabia and Tanzania. The former is clearly more affluent (even, I suspect, the population in the Eastern Saudi Arabia provinces where SCD is most common) and the latter has a higher mean per capita income compared to Uganda, so perhaps socio-economic circumstances might also play a role?  
12. Finally, as I suspect the authors know only too well with regards to their intended study of hydroxyurea in Uganda, the fact that it operates through "a degree of immunosuppression" has implications for starting a trial when those who are immunosuppressed may be being given advice to shield from COVID-19.

 

 I wish the authors every success with their future work.

Is the work clearly and accurately presented and does it cite the current literature?

Yes

If applicable, is the statistical analysis and its interpretation appropriate?

I cannot comment. A qualified statistician is required.

Are all the source data underlying the results available to ensure full reproducibility?

Yes

Is the study design appropriate and is the work technically sound?

Yes

Are the conclusions drawn adequately supported by the results?

Yes

Are sufficient details of methods and analysis provided to allow replication by others?

Yes

Reviewer Expertise:

Sociologist

I confirm that I have read this submission and believe that I have an appropriate level of expertise to confirm that it is of an acceptable scientific standard.

# Response

**Published:** 2020-05-05

**Authors:** Thomas N. Williams

**Affiliations:** KEMRI, Kenya

Thank you for these very helpful and constructive comments. Given that we anticipate receiving additional reviews we will defer our response until all reviews are in so the we can deal with the reviews holistically. 

Tom Williams on behalf of all authors
